# Revisiting Batch Normalization for Training Low-Latency Deep Spiking Neural Networks From Scratch

**DOI:** 10.3389/fnins.2021.773954

**Published:** 2021-12-09

**Authors:** Youngeun Kim, Priyadarshini Panda

**Affiliations:** Department of Electrical Engineering, Yale University, New Haven, CT, United States

**Keywords:** spiking neural network, batch normalization, image recognition, event-based processing, energy-efficient deep learning

## Abstract

Spiking Neural Networks (SNNs) have recently emerged as an alternative to deep learning owing to sparse, asynchronous and binary event (or spike) driven processing, that can yield huge energy efficiency benefits on neuromorphic hardware. However, SNNs convey temporally-varying spike activation through time that is likely to induce a large variation of forward activation and backward gradients, resulting in unstable training. To address this training issue in SNNs, we revisit Batch Normalization (BN) and propose a temporal Batch Normalization Through Time (BNTT) technique. Different from previous BN techniques with SNNs, we find that varying the BN parameters at every time-step allows the model to learn the time-varying input distribution better. Specifically, our proposed BNTT decouples the parameters in a BNTT layer along the time axis to capture the temporal dynamics of spikes. We demonstrate BNTT on CIFAR-10, CIFAR-100, Tiny-ImageNet, event-driven DVS-CIFAR10 datasets, and Sequential MNIST and show near state-of-the-art performance. We conduct comprehensive analysis on the temporal characteristic of BNTT and showcase interesting benefits toward robustness against random and adversarial noise. Further, by monitoring the learnt parameters of BNTT, we find that we can do temporal early exit. That is, we can reduce the inference latency by ~5 − 20 time-steps from the original training latency. The code has been released at https://github.com/Intelligent-Computing-Lab-Yale/BNTT-Batch-Normalization-Through-Time.

## 1. Introduction

Artificial Neural Networks (ANNs) have shown state-of-the-art performance across various computer vision tasks. Nonetheless, huge energy consumption incurred for implementing ANNs on conventional von-Neumann hardware limits their usage in low-power and resource-constrained Internet of Things (IoT) environment, such as mobile phones, drones among others. In the context of low-power machine intelligence, Spiking Neural Networks (SNNs) have received considerable attention in the recent past (Cao et al., 2015; Diehl and Cook, 2015; Roy et al., 2019; Comsa et al., 2020; Panda et al., 2020). Inspired by biological neuronal mechanisms, SNNs process visual information with discrete spikes or events over multiple time-steps. Recent works have shown that the event-driven behavior of SNNs can be implemented on emerging neuromorphic hardware to yield 1–2 order of magnitude energy efficiency over ANNs (Akopyan et al., 2015; Davies et al., 2018). Despite the energy efficiency benefits, SNNs have still not been widely adopted due to inherent training challenges. The training issue arises from the non-differentiable characteristic of a spiking neuron, generally, Integrate-and-Fire (IF) type (Burkitt, 2006), that makes SNNs incompatible with gradient descent training.

To address the training issue of SNNs, several methods, such as, *Conversion* and *Surrogate Gradient Descent* have been proposed. In ANN-SNN conversion (Diehl et al., 2015; Rueckauer et al., 2017; Sengupta et al., 2019; Han et al., 2020), off-the-shelf trained ANNs are converted to SNNs using normalization methods to transfer ReLU activation to IF spiking activity. The advantage here is that training happens in the ANN domain leveraging widely used machine learning frameworks like, PyTorch, that yield short training time and can be applied to complex datasets. But the ANN-SNN conversion method requires large number of time-steps (~ 500 − 1, 000) for inference to yield competitive accuracy, which significantly increases the latency and energy consumption of the SNN. On the other hand, directly training SNNs with a surrogate gradient function (Wu et al., 2018; Neftci et al., 2019; Lee et al., 2020) exploits temporal dynamics of spikes, resulting in lesser number of time-steps (~ 100 − 150). However, the discrepancy between forward spike activation function and backward surrogate gradient function during backpropagation restricts the training capability. Therefore, naive SNNs without additional optimization techniques are difficult to be trained on large-scale datasets (e.g., CIFAR-100 and Tiny-ImageNet). Recently, a hybrid method (Rathi et al., 2020) that combines the conversion method and the surrogate gradient-based method shows state-of-the-art performance at reasonable latency (~250 time-steps). However, the hybrid method incurs sequential processes, i.e., training ANN from scratch, conversion of ANN to SNN, and training SNNs using surrogate gradient descent, that increases the total computation cost to obtain the final SNN model. Overall, training high-accuracy and low-latency SNNs from scratch still remains an open problem.

In this paper, we investigate the temporal characteristics of Batch Normalization (BN) for more advanced SNN training. The BN layer (Ioffe and Szegedy, 2015) has been used extensively in deep learning to accelerate the training process of ANNs. It is well known that BN reduces internal covariate shift (or soothing optimization landscape Santurkar et al., 2018) mitigating the problem of exploding/vanishing gradients. In SNN literature, there are a few recent works that leverage BN layers during training and have shown competitive performance for image classification tasks with low latency. Ledinauskas et al. (2020) use a standard BN layer and show the scalability of SNNs toward deep architectures with BN layers. Fang et al. (2020) propose a learnable membrane time constant with a standard BN layer. Zheng et al. (2020) present the advantage of scaling BN parameter according to the neuronal firing threshold. Even though the previous BN approaches show performance/latency improvement, we assert that there is need to explore the advantage of BN in the temporal dimension since SNNs convey information through time. The previous BN works with SNNs use a single BN parameter across all time-steps. We are essentially motivated by the question, *Can a single learnable parameter in the BN layer learn the temporal characteristics of the input spikes that vary across different time-steps?*

**Figure 1:**
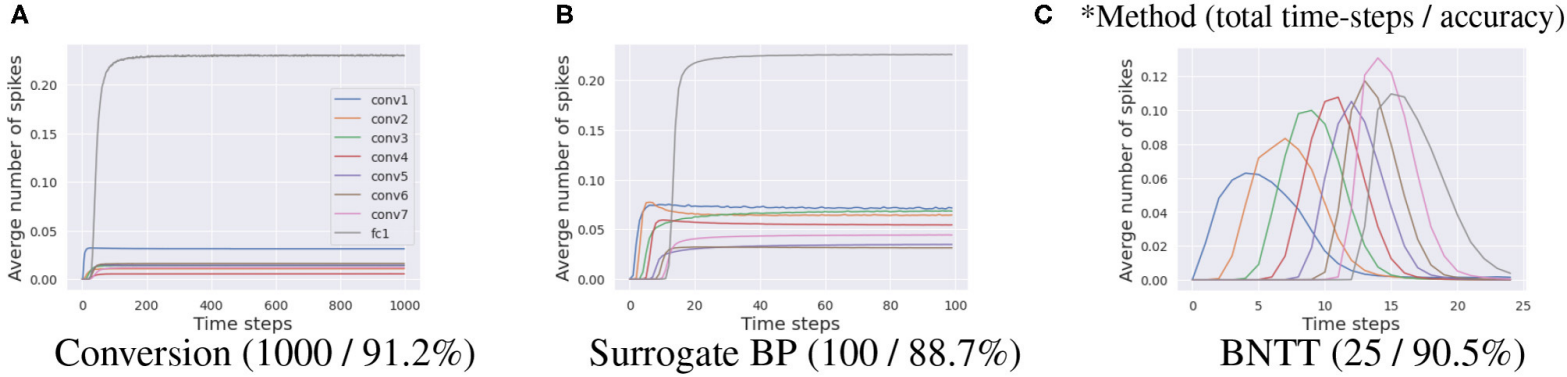
Visualization of the average number of spikes in each layer with respect to time-steps. Compared to **(A)** ANN-SNN conversion and **(B)** surrogate gradient-based backpropagation, our **(C)** BNTT captures the temporal dynamics of spike activation with learnable parameters, enabling low-latency (i.e., small time-steps) and low-energy (i.e., less number of spikes) training. All experiments are conducted on CIFAR-10 with VGG9.

Different from previous works, we highlight the importance of temporal characterization of BN technique. To this end, we propose a new SNN-crafted batch normalization layer called Batch Normalization Through Time (BNTT) that decouples the parameters in the BN layer across different time-steps. BNTT is implemented as an additional layer in SNNs and is trained with surrogate gradient backpropagation. To investigate the effect of our BNTT, we compare the statistics of spike activity of BNTT with previous approaches: Conversion (Sengupta et al., 2019) and standard Surrogate Gradient Descent (Neftci et al., 2019), as shown in Figure 1. Interestingly, different from the conversion method and surrogate gradient method (without BNTT) that maintain reasonable spike activity during the entire time period across different layers, spike activity of layers trained with BNTT follows a gaussian-like trend. BNTT imposes a variation in spiking across different layers, wherein, each layer's activity peaks in a particular time-step range and then decreases. Moreover, the peaks for early layers occur at initial time-steps and latter layers peak at later time-steps. This phenomenon implies that learnable parameters in BNTT enable the networks to pass the visual information temporally from shallow to deeper layers in an effective manner.

The newly observed characteristics of BNTT brings several advantages. First, similar to BN, the BNTT layer enables SNNs to be trained stably from scratch even for large-scale datasets. Second, learnable parameters in BNTT enable SNNs to be trained with low latency (~ 25 − 50 time-steps) and impose optimum spike activity across different layers for low-energy inference. Finally, the distribution of the BNTT learnable parameter (i.e., γ) is a good representation of the temporal dynamics of spikes. Hence, relying on the observation that low γ value induces low spike activity and vice-versa, we further propose a temporal early exit algorithm. Here, an SNN can predict at an earlier time-step and does not need to wait till the end of the time period to make a prediction.

In summary, our key contributions are as follows: (i) We explore the temporal characteristics of BN for SNNs and propose a temporally adaptive BN approach, called BNTT. (ii) BNTT allows SNNs to be implemented in a low-latency and low-energy environment. (iii) We further propose a temporal early exit algorithm at inference time by monitoring the learnable parameters in BNTT. (iv) To ascertain that BNTT captures the temporal characteristics of SNNs, we mathematically show that proposed BNTT has similar effect as controlling the firing threshold of the spiking neuron at every time step during inference.

## 2. Batch Normalization

Batch Normalization (BN) reduces the internal covariate shift (or variation of loss landscape Santurkar et al., 2018) caused by the distribution change of input signal, which is a known problem of deep neural networks (Ioffe and Szegedy, 2015). Instead of calculating the statistics of total dataset, the intermediate representations are standardized with a mini-batch to reduce the computation complexity. Given a mini-batch $B=\left\{x_{1,\ldots ,m}\right\}$, the BN layer computes the mean and variance of the mini-batch as:


(1) $$\begin{array}{l} \mu_{B}=\frac{1}{m}\overset{m}{\underset{b=1}{\sum }}x_{b};\;\sigma_{B}^{2}=\frac{1}{m}\overset{m}{\underset{b=1}{\sum }}{\left(x_{b}-\mu_{B}\right)}^{2}. \end{array}$$

Then, the input features in the mini-batch are normalized with calculated statistics as:


(2) $$\begin{array}{l} {\hat{x}}_{b}=\frac{x_{b}-\mu_{B}}{\sqrt{\sigma_{B}^{2}+\epsilon }}, \end{array}$$

where, ϵ is a small constant for numerical stability. To further improve the representation capability of the layer, learnable parameters γ and β are used to transform the input features that can be formulated as $BN\left(x_{i}\right)=\gamma {\hat{x}}_{i}+\beta$. At inference time, BN uses the running average of mean and variance obtained from training. In this work, different from the static BN, we explore the temporal characteristics of BN with SNNs by enabling temporally-varying parameters in BN.

## 3. Methodology

### 3.1. Spiking Neural Networks

Different from conventional ANNs, SNNs transmit information using binary spike trains. To leverage the temporal spike information, Leaky-Integrate-and-Fire (LIF) model (Dayan and Abbott, 2001) is widely used to emulate neuronal functionality in SNNs, which can be formulated as a differential equation:


(3) $$\begin{array}{l} \tau_{m}\frac{dU_{m}}{dt}=-U_{m}+RI\left(t\right), \end{array}$$

where, *U*_*m*_ represents the membrane potential of the neuron that characterizes the internal state of the neuron, τ_*m*_ is the time constant of membrane potential decay. Also, *R* and *I*(*t*) denote the input resistance and the input current at time *t*, respectively. Following the previous work (Wu et al., 2019), we convert this continuous dynamic equation into a discrete equation for digital simulation. For a single post-synaptic neuron *i*, we can represent the membrane potential $u_{i}^{t}$ at time-step *t* as:


(4) $$\begin{array}{l} u_{i}^{t}=\lambda u_{i}^{t-1}+\underset{j}{\sum }w_{ij}o_{j}^{t}. \end{array}$$

Here, *j* is the index of a pre-synaptic neuron, λ is a leak factor with value less than 1, *o*_*j*_ is the binary spike activation, and *w*_*ij*_ is the weight of the connection between pre- and post-neurons. From Equation (4), the membrane potential of a neuron decreases due to leak and increases due to the weighted sum of incoming input spikes.

**Figure 2:**
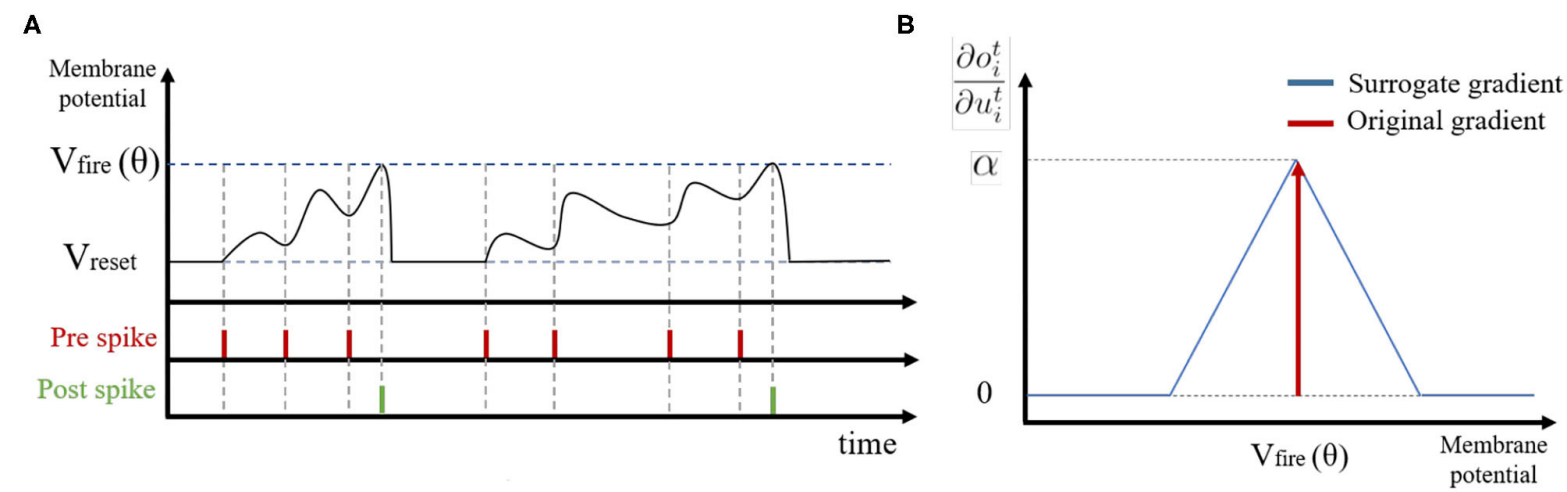
**(A)** Illustration of spike activities in Leaky-Integrate-and-Fire neurons. **(B)** The approximated gradient value with respect to the membrane potential.

If the membrane potential *u* exceeds a pre-defined firing threshold θ, the LIF neuron *i* generates a binary spike output *o*_*i*_. After that, we perform a soft reset, where the membrane potential *u*_*i*_ is reset by reducing its value by the threshold θ. Compared to a hard reset (resetting the membrane potential *u*_*i*_ to zero after neuron *i* spikes), the soft reset minimizes information loss by maintaining the residual voltage and carrying it forward to the next time step, thereby achieving better performance (Han et al., 2020). Figure 2A illustrates the membrane potential dynamics of a LIF neuron.

For the output layer, we discard the thresholding functionality so that neurons do not generate any spikes. We allow the output neurons to accumulate the spikes over all time-steps by fixing the leak parameter (λ in Equation 4) as one. This enables the output layer to compute probability distribution after softmax function without information loss. As with ANNs, the number of output neurons in SNNs is identical to the number of classes *C* in the dataset. From the accumulated membrane potential, we can define the cross-entropy loss for SNNs as:


(5) $$\begin{array}{l} L=-\underset{i}{\sum }y_{i}log\left(\frac{e^{u_{i}^{T}}}{\sum_{k=1}^{C}e^{u_{k}^{T}}}\right), \end{array}$$

where, *y* is the ground-truth label, and *T* represents the total number of time-steps. Then, the weights of all layers are updated by backpropagating the loss value with gradient descent.

To compute the gradients of each layer *l*, we use back-propagation through time (BPTT), which accumulates the gradients over all time-steps (Wu et al., 2018; Neftci et al., 2019). These approaches can be implemented with auto-differentiation tools, such as PyTorch (Paszke et al., 2017), that enable backpropagation on the unrolled network. To this end, we compute the loss function at time-step *T* and use gradient descent optimization. Mathematically, we can define the accumulated gradients at the layer *l* by chain rule as:


(6) $$\begin{array}{l} \frac{\partial L}{\partial W_{l}}=\{\begin{array}{cc} \sum_{t}\left(\frac{\partial L}{\partial O_{l}^{t}}\frac{\partial O_{l}^{t}}{\partial U_{l}^{t}}+\frac{\partial L}{\partial U_{l}^{t+1}}\frac{\partial U_{l}^{t+1}}{\partial U_{l}^{t}}\right)\frac{\partial U_{l}^{t}}{\partial W_{l}}, & \text{if }l\text{= hidden layer}\\ \sum_{t}\frac{\partial L}{\partial U_{l}^{T}}\frac{\partial U_{l}^{T}}{\partial W_{l}}. & \text{if }l\text{= output layer} \end{array} \end{array}$$

Here, *O*_*l*_ and *U*_*l*_ are output spikes and membrane potential at layer *l*, respectively. For the output layer, we get the derivative of the loss *L* with respect to the membrane potential $u_{i}^{T}$ at final time-step *T*:


(7) $$\begin{array}{l} \frac{\partial L}{\partial u_{i}^{T}}=\frac{e^{u_{i}^{T}}}{\sum_{k=1}^{C}e^{u_{k}^{T}}}-y_{i}. \end{array}$$

This derivative function is continuous and differentiable for all possible membrane potential values.

On the other hand, LIF neurons in hidden layers generate spike output only if the membrane potential $u_{i}^{t}$ exceeds the firing threshold, leading to non-differentiability. To deal with this problem, we introduce an approximate gradient (Figure 2B):


(8) $$\begin{array}{l} \frac{\partial o_{i}^{t}}{\partial u_{i}^{t}}=\alpha \text{ max}\left\{0,1-\;|\frac{u_{i}^{t}-\theta }{\theta }\;|\right\}, \end{array}$$

where, α is a damping factor for back-propagated gradients. Note, a large α value causes unstable training as gradients are summed over all time-steps. Hence, we set α to 0.3. Overall, we update the network parameters at the layer *l* based on the gradient value (Equation 6) as *W*_*l*_ = *W*_*l*_ − ηΔ*W*_*l*_.

### 3.2. Batch Normalization Through Time (BNTT)

**Figure 3:**
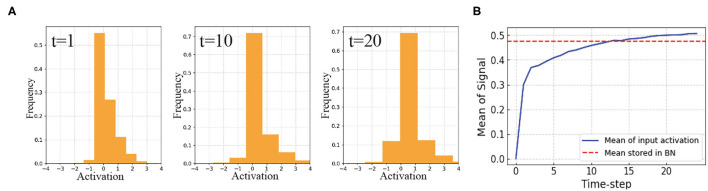
SNNs with standard BN: **(A)** Distributions of the input activation of BN at time-step 1, 10, and 20. **(B)** While the mean of input activation varies with time, stored mean in standard BN layer has constant value at inference. This will create discrepancy and inhibit the BN layer to learn well. This suggests a temporally varying BN technique.

In this work, we present a new temporally-variant Batch Normalization for accelerating SNN training. We first visualize the distribution of the input signal of standard BN at layer 5 in VGG9 SNN with surrogate-gradients based training (Figure 3). The results show that the input signal to the BN layer varies with time. Therefore, we assert that if we enable temporal flexibility to BN parameters (e.g., global mean μ, global variation σ, and learnable parameter γ), the representation power of the networks might be improved.

To this end, we vary the internal parameters in a BN layer through time, that we define as, BNTT. Similar to the digital simulation of LIF neuron across different time-steps, one BNTT layer is expanded temporally with a local learning parameter associated with each time-step. This allows the BNTT layer to capture temporal statistics (see section 3.3 for mathematical analysis). The proposed BNTT layer is easily applied to SNNs by inserting the layer after convolutional/linear operations as:


(9) $$\begin{array}{l} u_{i}^{t}=\lambda u_{i}^{t-1}+BNTT_{\gamma^{t}}\left(\underset{j}{\sum }w_{ij}o_{j}^{t}\right)\\ \;=\lambda u_{i}^{t-1}+\gamma_{i}^{t}\left(\frac{\sum_{j}w_{ij}o_{j}^{t}-\mu_{i}^{t}}{\sqrt{{\left(\sigma_{i}^{t}\right)}^{2}+\epsilon }}\right). \end{array}$$

During the training process, we compute the mean $\mu_{i}^{t}$ and variance $\sigma_{i}^{t}$ from the samples in a mini-batch B for each time step *t*, as shown in Algorithm 1. Note, for each time-step *t*, we apply an exponential moving average to approximate global mean ${\overset{-}{\mu }}_{i}^{t}$ and variance ${\overset{-}{\sigma }}_{i}^{t}$ over training iterations. These global statistics are used to normalize the test data at inference. Also, we do not utilize β as in conventional BN, since it adds redundant voltage to the membrane potential of SNNs.

**Algorithm 1:** BNTT layer

**Input:** mini-batch B at time step *t* ($x_{\left\{1\ldots m\right\}}^{t}$), learnable parameter (γ^*t*^), update factor (α)
**Output:** $\left\{y^{t}={\text{BNTT}}_{\gamma^{t}}\left(x^{t}\right)\right\}$
1: $\mu^{t}\leftarrow \frac{1}{m}\overset{m}{\underset{b=1}{\sum }}x_{b}^{t}$
2: ${\left(\sigma^{t}\right)}^{2}\leftarrow \frac{1}{m}\overset{m}{\underset{b=1}{\sum }}{\left(x_{b}^{t}-\mu^{t}\right)}^{2}$
3: ${\hat{x}}^{t}=\frac{x^{t}-\mu^{t}}{\sqrt{{\left(\sigma^{t}\right)}^{2}+\epsilon }}$
4: $y^{t}\leftarrow \gamma^{t}{\hat{x}}^{t}\equiv {\text{BNTT}}_{\gamma^{t}}\left(x^{t}\right)$
5: % Exponential moving average
6: ${\overset{-}{\mu }}^{t}\leftarrow \left(1-\alpha \right){\overset{-}{\mu }}^{t}+\alpha \mu^{t}$
7: ${\overset{-}{\sigma }}^{t}\leftarrow \left(1-\alpha \right){\overset{-}{\sigma }}^{t}+\alpha \sigma^{t}$

Adding the BNTT layer to LIF neurons changes the gradient calculation for backpropagation. Given that $x_{i}^{t}=\sum_{j}w_{ij}o_{j}^{t}$ is an input signal to the BNTT layer, we can calculate the gradient value passed through lower layers by the BNTT layer as:


(10) $$\begin{array}{l} \frac{\partial L}{\partial x_{b}^{t}}=\frac{1}{m\sqrt{{\left(\sigma^{t}\right)}^{2}+\epsilon }}\left(m\frac{\partial L}{\partial {\hat{x}}_{b}^{t}}-\overset{m}{\underset{k=1}{\sum }}\frac{\partial L}{\partial {\hat{x}}_{k}^{t}}-{\hat{x}}_{b}^{t}\overset{m}{\underset{k=1}{\sum }}\frac{\partial L}{\partial {\hat{x}}_{k}^{t}}{\hat{x}}_{k}^{t}\right). \end{array}$$

Here, we omit a neuron index *i* for simplicity. Also, *m* and *b* denote the batch size and batch index (see Supplementary Material A for more detail). Thus, for every time-step *t*, gradients are calculated based on the time-specific statistics of input signals. This allows the networks to take into account temporal dynamics for training weight connections. Moreover, a learnable parameter γ is updated to restore the representation power of the batch normalized signal. Since we use different γ^*t*^ values across all time-steps, γ^*t*^ finds an optimum over each time-step for efficient inference. We update gamma γ^*t*^ = γ^*t*^ − ηΔγ^*t*^ where:


(11) $$\begin{array}{l} \Delta \gamma^{t}=\frac{\partial L}{\partial \gamma^{t}}=\frac{\partial L}{\partial u^{t}}\frac{\partial u^{t}}{\partial \gamma^{t}}=\overset{m}{\underset{k=1}{\sum }}\frac{\partial L}{\partial u_{k}^{t}}{\hat{x}}_{k}^{t}. \end{array}$$

### 3.3. Mathematical Analysis

In this section, we discuss the connections between BNTT and the firing threshold of a LIF neuron. Specifically, we formally prove that using BNTT has a similar effect as varying the firing threshold over different time-steps, thereby ascertaining that BNTT captures temporal characteristics in SNNs. Recall that BNTT normalizes the input signal using stored approximated global average ${\overset{-}{\mu }}_{i}^{t}$ and standard deviation ${\left({\overset{-}{\sigma_{i}}}^{t}\right)}^{2}$ at inference. From Equation (9), we can calculate a membrane potential at time-step *t* = 1, given that initial membrane potential $u_{i}^{0}$ has a zero value:


(12) $$\begin{array}{l} u_{i}^{1}=\gamma_{i}^{1}\left(\frac{\sum_{j}w_{ij}o_{j}^{1}-{\overset{-}{\mu }}_{i}^{1}}{\sqrt{{\left({\overset{-}{\sigma }}_{i}^{1}\right)}^{2}+\epsilon }}\right)\\ \;\approx \frac{\gamma_{i}^{1}}{\sqrt{{\left({\overset{-}{\sigma }}_{i}^{1}\right)}^{2}+\epsilon }}\underset{j}{\sum }w_{ij}o_{j}^{1}=\frac{\gamma_{i}^{1}}{\sqrt{{\left({\overset{-}{\sigma }}_{i}^{1}\right)}^{2}+\epsilon }}{ũ}_{i}^{1}. \end{array}$$

Here, we assume ${\overset{-}{\mu }}_{i}^{1}$ can be neglected with small signal approximation due to the spike sparsity in SNNs, and ${ũ}_{i}^{1}=\sum_{j}w_{ij}o_{j}^{1}$ is membrane potential at time-step *t* = 1 without BNTT (obtained from Equation 4). We can observe that the membrane potential with BNTT is proportional to the membrane potential without BNTT at *t* = 1. For time-step *t* > 1, we should take into account the membrane potential from the previous time-step, which is multiplied by leak λ. To this end, by substituting (Equation 12) in the BNTT equation (Equation 9), we can formulate the membrane potential at *t* = 2 as:


(13) $$\begin{array}{l} u_{i}^{2}\approx \lambda u_{i}^{1}+\frac{\gamma_{i}^{2}}{\sqrt{{\left(\sigma_{i}^{2}\right)}^{2}+\epsilon }}\underset{j}{\sum }w_{ij}o_{j}^{2}\\ \;=\left(\frac{\lambda \gamma_{i}^{1}}{\sqrt{{\left(\sigma_{i}^{1}\right)}^{2}+\epsilon }}\right){ũ}_{i}^{1}+\frac{\gamma_{i}^{2}}{\sqrt{{\left(\sigma_{i}^{2}\right)}^{2}+\epsilon }}\underset{j}{\sum }w_{ij}o_{j}^{2}\\ \;\approx \frac{\gamma_{i}^{2}}{\sqrt{{\left(\sigma_{i}^{2}\right)}^{2}+\epsilon }}\left\{\lambda {ũ}_{i}^{1}+\underset{j}{\sum }w_{ij}o_{j}^{2}\right\}=\frac{\gamma_{i}^{2}}{\sqrt{{\left(\sigma_{i}^{2}\right)}^{2}+\epsilon }}{ũ}_{i}^{2}. \end{array}$$

In the third line, the learnable parameter $\gamma_{i}^{t}$ and $\sigma_{i}^{t}$ have similar values in adjacent time intervals (*t* = 1, 2) because of continuous time property. Hence, we can approximate $\gamma_{i}^{1}$ and $\sigma_{i}^{1}$ as $\gamma_{i}^{2}$ and $\sigma_{i}^{2}$, respectively. Finally, we can extend the equation of BNTT to the time-step *t*:


(14) $$\begin{array}{l} u_{i}^{t}\approx \frac{\gamma_{i}^{t}}{\sqrt{{\left(\sigma_{i}^{t}\right)}^{2}+\epsilon }}{ũ}_{i}^{t}. \end{array}$$

Considering that a neuron produces an output spike activation whenever the membrane potential ${ũ}_{i}^{t}$ exceeds the pre-defined firing threshold θ, the spike firing condition with BNTT can be represented $u_{i}^{t}\geq \theta$. Comparing with the threshold of a neuron without BNTT, we can reformulate the firing condition as:


(15) $$\begin{array}{l} {ũ}_{i}^{t}\geq \frac{\sqrt{{\left(\sigma_{i}^{t}\right)}^{2}+\epsilon }}{\gamma_{i}^{t}}\theta . \end{array}$$

Thus, we can infer that using a BNTT layer changes the firing threshold value by $\sqrt{{\left(\sigma_{i}^{t}\right)}^{2}+\epsilon }/\gamma_{i}^{t}$ at every time-step. In practice, BNTT results in an optimum γ during training that improves the representation power, producing better performance and low-latency SNNs. This observation allows us to consider the advantages of time-varying learnable parameters in SNNs. This implication is in line with previous work (Han et al., 2020), which insists that manipulating the firing threshold improves the performance and latency of the ANN-SNN conversion method. However, Han et al. change the threshold value in a heuristic way without any optimization process and fix the threshold value across all time-steps. On the other hand, our BNTT yields time-specific γ^*t*^ which can be optimized via back-propagation.

### 3.4. Early Exit Algorithm

The main objective of early exit is to reduce the latency during inference (Panda et al., 2016; Teerapittayanon et al., 2016). Most previous methods (Wu et al., 2018; Sengupta et al., 2019; Han et al., 2020; Lee et al., 2020; Rathi et al., 2020) accumulate output spikes till the end of the time-sequence, at inference, since all layers generate spikes across all time-steps as shown in Figures 1A,B. On the other hand, learnable parameters in BNTT manipulate the spike activity of each layer to produce a peak value, which falls again (a gaussian-like trend), as shown in Figure 1C. This phenomenon shows that SNNs using BNTT convey little information at the end of spike trains.

**Figure 4:**
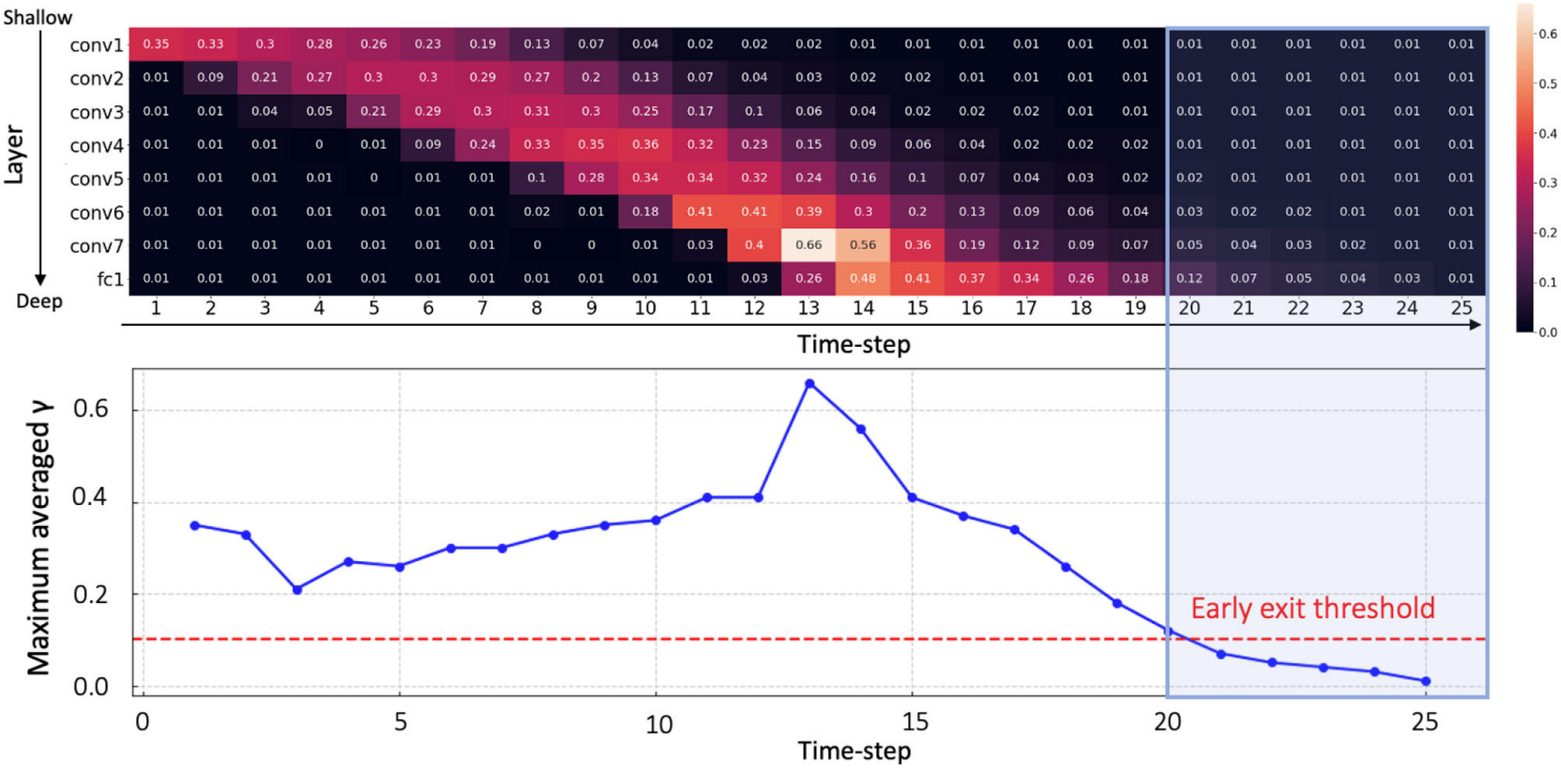
The average value of γ^*t*^ at each layer over all time-steps (upper panel). Maximum averaged γ^*t*^ for each time-step (lower panel). Early exit time can be calculated as *t* = 20 since γ^*t*^ values at every layer have lower value than threshold 0.1 after time-step 20 (blue shaded area). Here, we use a VGG9 architecture on CIFAR-10.

Inspired by this observation, we propose a temporal early exit algorithm based on the value of γ^*t*^. From Equation (15), we know that a low γ^*t*^ value increases the firing threshold, resulting in low spike activity. A high γ^*t*^ value, in contrast, induces more spike activity. It is worth mentioning that ${\left(\sigma_{i}^{t}\right)}^{2}$ shows similar values across all time-steps and therefore we only focus on γ^*t*^. Given that the intensity of spike activity is proportional to γ^*t*^, we can infer that spikes will hardly contribute to the classification result once γ^*t*^ values across every layer drop to a minimum value. Therefore, we measure the average of γ^*t*^ values in each layer *l* at every time-step, and terminate the inference when γ^*t*^ value in every layer is below a pre-determined threshold. For example, as shown in Figure 4, we observe that all averaged γ^*t*^ values are lower than threshold 0.1 after *t* > 20. Therefore, we define the early exit time at *t* = 20. Note that we can determine the optimum time-step for early exit before forward propagation without any additional computation. In summary, the temporal early exit method enables us to find the earliest time-step during inference that ensures integration of crucial information, in turn reducing the inference latency without significant loss of accuracy.

### 3.5. Overall Optimization

Algorithm 2 summarizes the whole training process of SNNs with BNTT. Our proposed BNTT acts as a regularizer, unlike previous methods (Lee et al., 2016, 2020; Sengupta et al., 2019; Rathi et al., 2020) that use dropout to perform regularization. Our training scheme is based on widely used rate coding where the spike generator produces a Poisson spike train (see Supplementary Material B) for each pixel in the image with frequency proportional to the pixel intensity (Roy et al., 2019). For all layers, the weighted sum of the input signal is passed through a BNTT layer and then is accumulated in the membrane potential. If the membrane potential exceeds the firing threshold, the neuron generates an output spike. For last layer, we accumulate the input voltage over all time-steps without leak, that we feed to a softmax layer to output a probability distribution. Then, we calculate a cross-entropy loss function and gradients for weight of each layer with the approximate gradient function. During the training phase, a BNTT layer computes the time-dependent statistics (i.e., μ^*t*^ and σ^*t*^) and stores the moving-average global mean and variance. At inference, we first define the early exit time-step based on the value of γ in BNTT. Then, the networks classify the test input (note, test data normalized with pre-computed global ${\overset{-}{\mu }}^{t},{\overset{-}{\sigma }}^{t}$ BNTT statistics) based on the accumulated output voltage at the pre-computed early exit time-step.

**Algorithm 2:** Training process with BNTT

**Input:** mini-batch (*X*); label set (*Y*); max_timestep (*T*)
**Output:** updated network weights
1: **for** *i* ← 1 to *max*_*iter* **do**
2: fetch a mini batch X
3: **for** *t* ← 1 to *T* **do**
4: O ← PoissonGenerator(X)
5: **for** *l* ← 1 to *L*−1 **do**
6: $\left(O_{l}^{t},U_{l}^{t}\right)\leftarrow \left(\lambda ,U_{l}^{t-1},{\text{BNTT}}_{\gamma^{t}}\left(W_{l},O_{l-1}^{t-1}\right)\right)$
7: **end for**
8: % For the final layer *L*, stack the voltage
9: $U_{L}^{t}\leftarrow \left(U_{L}^{t-1},{\text{BNTT}}_{\gamma^{t}}\left(W_{l},O_{L-1}^{t-1}\right)\right)$
10: **end for**
11: % Calculate the loss and back-propagation
12: $L\leftarrow \left(U_{L}^{T},Y\right)$
13: **end for**

## 4. Experiments

In this section, we carry out comprehensive experiments on public classification datasets. We first compare our BNTT with previous SNNs training methods. Then, we quantitatively and qualitatively demonstrate the effectiveness of our proposed BNTT.

### 4.1. Experimental Setup

We evaluate our method on three static datasets (i.e., CIFAR-10, CIFAR-100, Tiny-ImageNet), one neuromophic dataset (i.e., DVS-CIFAR10), and one sequential dataset (i.e., Sequential MNIST). **CIFAR-10** (Krizhevsky and Hinton, 2009) consists of 60,000 images (50,000 for training/10,000 for testing) with 10 categories. All images are RGB color images whose size are 32 × 32. **CIFAR-100** has the same configuration as CIFAR-10, except it contains images from 100 categories. **Tiny-ImageNet** is the modified subset of the original ImageNet dataset. Here, there are 200 different classes of ImageNet dataset (Deng et al., 2009), with 100,000 training and 10,000 validation images. The resolution of the images is 64 × 64 pixels. **DVS-CIFAR10** (Li et al., 2017) has the same configuration as CIFAR-10. This discrete event-stream dataset is collected by moving the event-driven camera. We follow the similar data pre-processing protocol and a network architecture used in previous work (Wu et al., 2019) (details in Supplementary Material C). **Sequential MNIST** (Le et al., 2015) is the variant of MNIST (LeCun et al., 1998). Instead of showing the whole image to the networks, this dataset presents each pixel in an image pixel by pixel. Our implementation is based on Pytorch (Paszke et al., 2017). We train the networks with standard SGD with momentum 0.9, weight decay 0.0005 and also apply random crop and horizontal flip to input images. The base learning rate is set to 0.3 and we use step-wise learning rate scheduling with a decay factor 10 at 50, 70, and 90% of the total number of epochs. Here, we set the total number of epochs to 120, 240, 90, and 60 for CIFAR-10, CIFAR-100, Tiny-ImageNet, and DVS-CIFAR10, respectively.

### 4.2. Comparison With Previous Methods

On public datasets, we compare our proposed BNTT method with previous rate-coding based SNN training methods, including ANN-SNN conversion (Cao et al., 2015; Sengupta et al., 2019; Han et al., 2020), surrogate gradient back-propagation (Lee et al., 2020), and hybrid (Rathi et al., 2020) methods. From Table 1, we can observe some advantages and disadvantages of each training method. The ANN-SNN conversion method performs better than the surrogate gradient method across all datasets. However, they require large number of time-steps for training and testing, which is energy-inefficient and impractical in a real-time application. The hybrid method aims to resolve this high-latency problem, but it still requires over hundreds of time-steps. The surrogate gradient method (denoted as *Baseline*) suffers from poor optimization and hence cannot be scaled to larger datasets such as CIFAR-100 and Tiny-ImageNet. The results show that the performance improvement of SNN models is because of BNTT, and not because of applying the loss to the membrane potential which can improve the performance of SNNs (Eshraghian et al., 2021). Using standard BN with surrogate gradient training (i.e., *Baseline* + *standard BN*) improves the optimization capability of SNNs enabling us to train deep SNNs for complex datasets, however, there is performance degradation. Increasing the number of time-steps to > 100 − 150 does improve the performance, but that would also lead to increased computation. Our BNTT is based on the surrogate gradient method (i.e., Baseline + BNTT), and it enables SNNs to achieve high performance even for more complicated datasets. At the same time, we reduce the latency due to the inclusion of learnable parameters and temporal statistics in the BNTT layer. As a result, BNTT can be trained with 25 time-steps on a simple CIFAR-10 dataset, while preserving state-of-the-art accuracy. For CIFAR-100, we achieve about 40× and 2× faster inference speed compared to the conversion methods and the hybrid method, respectively. Interestingly, for Tiny-ImageNet, BNTT achieves better performance and shorter latency compared to previous conversion method. Note that ANN with VGG11 architecture used for ANN-SNN conversion achieves 56.3% accuracy. Moreover, using an early exit algorithm further reduces the latency by ~20%, which enables the networks to be implemented with lower-latency and energy-efficiency. It is worth mentioning that surrogate gradient method without BNTT (*Baseline* in Table 1) only converges on CIFAR-10. For neuromorphic DVS-CIFAR10 dataset (Table 2), using BNTT improves the stability of training compared to a surrogate gradient baseline, and achieves state-of-the-art performance. These results show that our BNTT technique is very effective on event-driven data and hence well-suited for neuromorphic applications. We also compare the performance of BNTT with previous works on Sequential MNIST in Table 3. Here, we use 3-layer SNN architecture: FC(1,256)-FC(256,256)-FC(256,10). Without BNTT, *Baseline* has difficulty in capturing the sequential pattern of input data, resulting in low performance. Adding BNTT to *Baseline* enhances the training capability of SNNs, resulting in a slightly better performance than the state-of-the-art (Bellec et al., 2018).

**Table 1:** Classification accuracy (%) on CIFAR-10, CIFAR-100, and Tiny-ImageNet.

	**Dataset**	**Training method**	**Architecture**	**Time-steps**	**Accuracy (%)**
Cao et al. (2015)	CIFAR-10	ANN-SNN Conversion	3Conv, 2Linear	400	77.4
Sengupta et al. (2019)	CIFAR-10	ANN-SNN Conversion	VGG16	2500	91.5
Lee et al. (2020)	CIFAR-10	Surrogate Gradient	VGG9	100	90.4
Rathi et al. (2020)	CIFAR-10	Hybrid	VGG16	200	92.0
Han et al. (2020)	CIFAR-10	ANN-SNN Conversion	VGG16	2048	93.6
Baseline	CIFAR-10	Surrogate Gradient	VGG9	100	88.7
Baseline + standard BN	CIFAR-10	Surrogate Gradient	VGG9	25	84.3
**Baseline + BNTT (ours)**	CIFAR-10	Surrogate Gradient	VGG9	25	90.5
**Baseline + BNTT + Early Exit (ours)**	CIFAR-10	Surrogate Gradient	VGG9	20	90.3
Sengupta et al. (2019)	CIFAR-100	ANN-SNN Conversion	VGG16	2500	70.9
Rathi et al. (2020)	CIFAR-100	Hybrid	VGG16	125	67.8
Han et al. (2020)	CIFAR-100	ANN-SNN Conversion	VGG16	2048	70.9
Baseline	CIFAR-100	Surrogate Gradient	VGG11	n/a	n/a
Baseline + standard BN	CIFAR-100	Surrogate Gradient	VGG11	50	43.0
**Baseline + BNTT (ours)**	CIFAR-100	Surrogate Gradient	VGG11	50	66.6
**Baseline + BNTT + Early Exit (ours)**	CIFAR-100	Surrogate Gradient	VGG11	30	65.8
Sengupta et al. (2019)	Tiny-ImageNet	ANN-SNN Conversion	VGG11	2500	54.2
Baseline	Tiny-ImageNet	Surrogate Gradient	VGG11	n/a	n/a
Baseline + standard BN	Tiny-ImageNet	Surrogate Gradient	VGG11	30	32.7
**Baseline + BNTT (ours)**	Tiny-ImageNet	Surrogate Gradient	VGG11	30	57.8
**Baseline + BNTT + Early Exit (ours)**	Tiny-ImageNet	Surrogate Gradient	VGG11	25	56.8

**Table 2:** Classification accuracy (%) on DVS-CIFAR10.

**Method**	**Type**	**Accuracy (%)**
Orchard et al. (2015)	Random forest	31.0
Lagorce et al. (2016)	HOTS	27.1
Sironi et al. (2018)	HAT	52.4
Sironi et al. (2018)	Gabor-SNN	24.5
Wu et al. (2019)	Surrogate gradient	60.5
Baseline	Surrogate gradient	n/a
**Baseline + BNTT (ours)**	Surrogate gradient	63.2

**Table 3:** Classification accuracy (%) on sequential MNIST.

**Method**	**Accuracy (%)**
LIF (Bellec et al., 2018)	63.3
LSNN (Bellec et al., 2018)	93.7
DEEP R LSNN (Bellec et al., 2018)	96.4
Baseline	36.2
**Baseline + BNTT (ours)**	96.6

### 4.3. Comparison With the Previous BN Techniques for SNNs

We compare our temporal BNTT technique with the previous BN approaches for SNN in Table 4. The approaches with the standard BN (Fang et al., 2020; Ledinauskas et al., 2020) do not show scalability to complicated datasets such as CIFAR-100 and Tiny-ImageNet. Compared to this, our approach enables training SNNs with low latency on such datasets. Zheng et al. (2020) show the advantage of scaling BN parameter according to the firing threshold, which shows good performance for large-scale datasets, including ImageNet. Our objective is to study the effect of BN in temporal domain, not enhance the capability of BN itself, which is different from their approach. Combining these two orthogonal approaches in order to achieve further performance gain can be a good topic for future work.

**Table 4:** Comparison between different BN techniques for SNNs.

	**Method**	**CIFAR-10**	**CIFAR-100**	**Tiny-imageNet**	**ImageNet**
Ledinauskas et al. (2020)	Standard BN	90.2	58.5	-	-
Fang et al. (2020)	Standard BN	93.5	-	-	-
Zheng et al. (2020)	Threshold-dependent BN	93.2	-	-	67.1
BNTT (ours)	Temporal BN	90.5	66.6	57.8	-

### 4.4. Spike Activity Analysis

**Figure 5:**
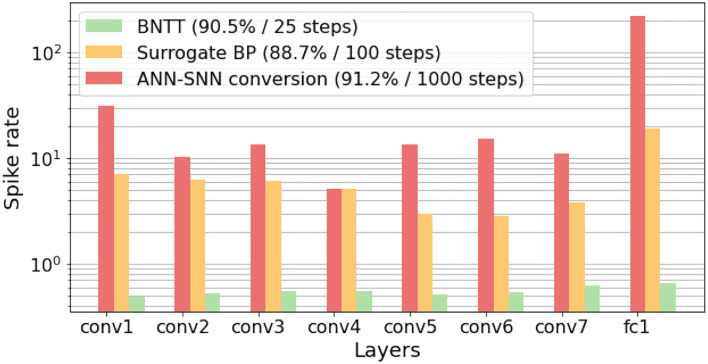
Visualization layer-wise spike activity (log scale) in VGG9 on CIFAR-10 dataset.

We compare the layer-wise spiking activities of our BNTT with two widely-used methods, i.e., ANN-SNN conversion method (Sengupta et al., 2019) and surrogate gradient method (without BNTT) (Neftci et al., 2019). Specifically, we calculate the spike rate of each layer *l*, which can be defined as the total number of spikes at layer *l* over total time-steps *T* divided by the number of neurons in layer *l* (see Supplementary Material D for the equation of spike rate). In Figure 5, converted SNNs show a high spike rate for every layer as they forward spike trains through a larger number of time-steps compared to other methods. Even though the surrogate gradient method uses less number of time-steps, it still requires nearly hundreds of spikes for each layer. Compared to these methods, we can observe that BNTT significantly improves the spike sparsity across all layers. In addition, we conduct further energy comparison on Neuromorphic architecture in Supplementary Material E.

### 4.5. Analysis on Learnable Parameters in BNTT

**Figure 6:**
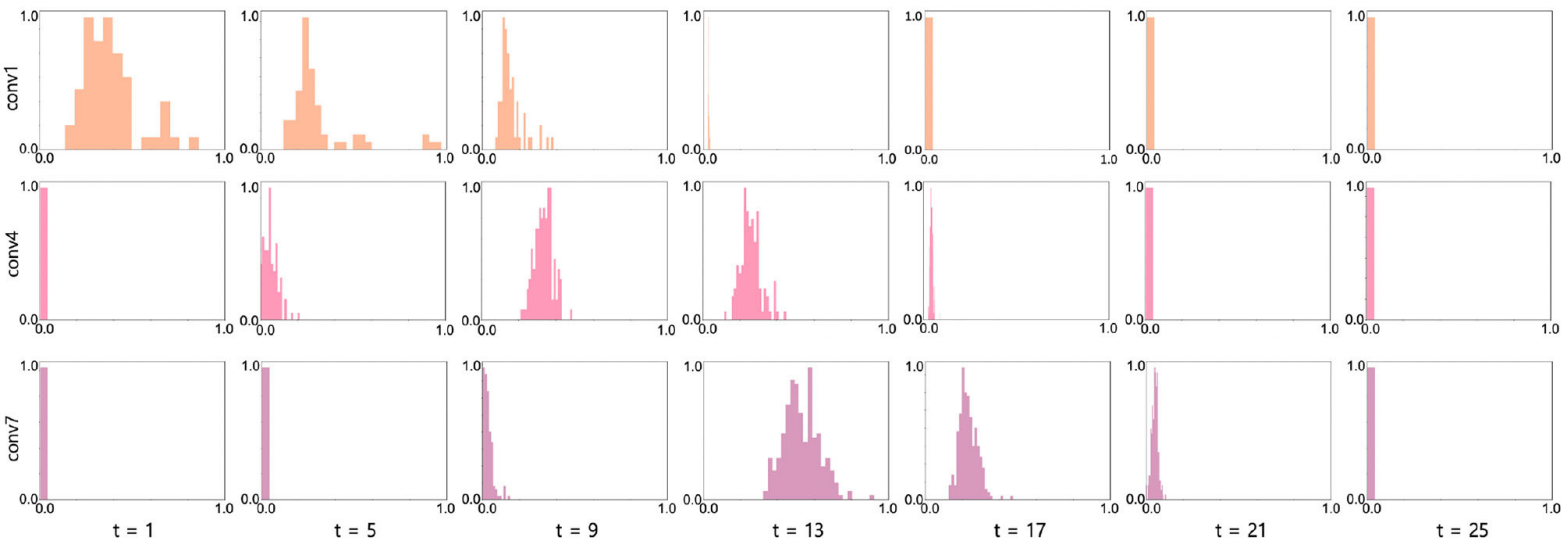
Histogram visualization (x axis: γ value, y axis: frequency) at conv1 (row1), conv4 (row2), and conv7 (row3) layers in VGG9 across all time-steps. We normalize the frequency into range [0, 1] for better visualization. The experiments are conducted on CIFAR-10 with 25 time-steps.

The key observation of our work is the change of γ across time-steps. To analyze the distribution of the learnable parameters in our BNTT, we visualize the histogram of γ in conv1, conv4, and conv7 layers in VGG9 as shown in Figure 6. Interestingly, all layers show different temporal evolution of gamma distributions. For example, conv1 has high γ values at the initial time-steps which decrease as time goes on. On the other hand, starting from small values, the γ values in conv4 and conv7 layers peak at *t* = 9 and *t* = 13, respectively, and then shrink to zero at later time-steps. Notably, the peak time is delayed as the layer goes deeper, implying that the visual information is passed through the network sequentially over a period of time similar to Figure 1C. This gaussian-like trend with rise and fall of γ across different time-steps can support the explanation of overall low spike activity compared to other methods (Figure 5).

### 4.6. Analysis on Early Exit

**Figure 7:**
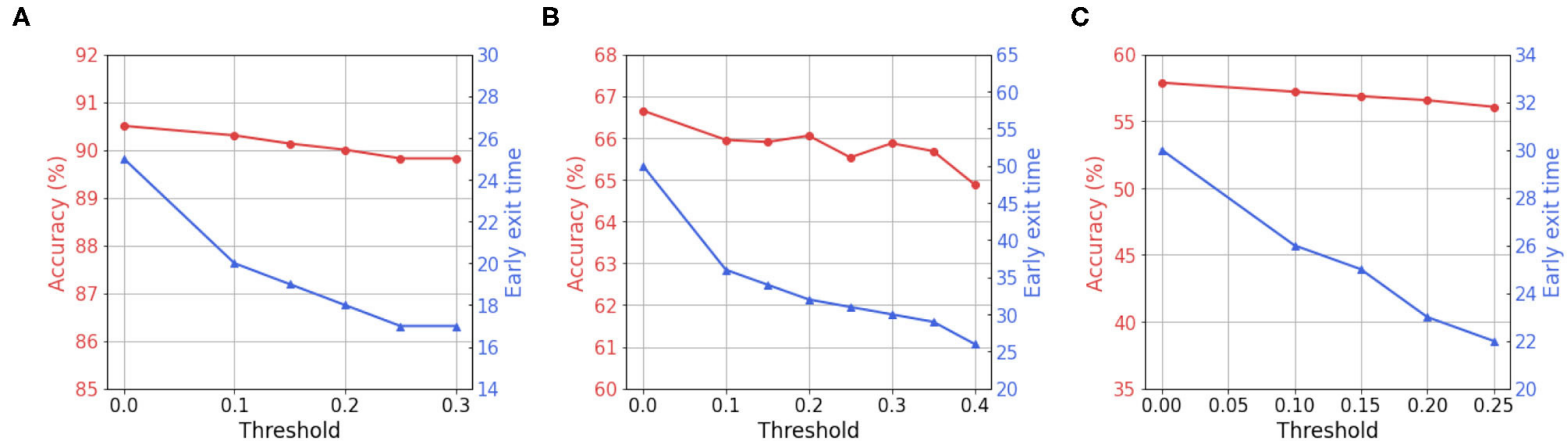
Visualization of accuracy and early exit time with respect to the threshold value for γ. **(A)** CIFAR-10. **(B)** CIFAR-100. **(C)** Tiny-ImageNet.

Recall that we measure the average of γ values in each layer at every time-step, and stop the inference when all γ values in every layer is lower than a predetermined threshold. To further investigate this, we vary the predetermined threshold and show the accuracy and exit time T_*exit*_ trend. As shown in Figure 7, we observe that high threshold enables the networks to infer at earlier time-steps. Although we use less number time-steps during inference, the accuracy drops marginally. This implies that BNTT rarely sends crucial information at the end of spike train (see Figure 1C). Note that the temporal evolution of learnable parameter γ with our BNTT allows us to exploit the early exit algorithm that yields a huge advantage in terms of reduced latency at inference. Such strategy has not been proposed or explored in any prior works that have mainly focused on reducing the number of time-steps during training without effectively using temporal statistics.

### 4.7. Analysis on Robustness

**Figure 8:**
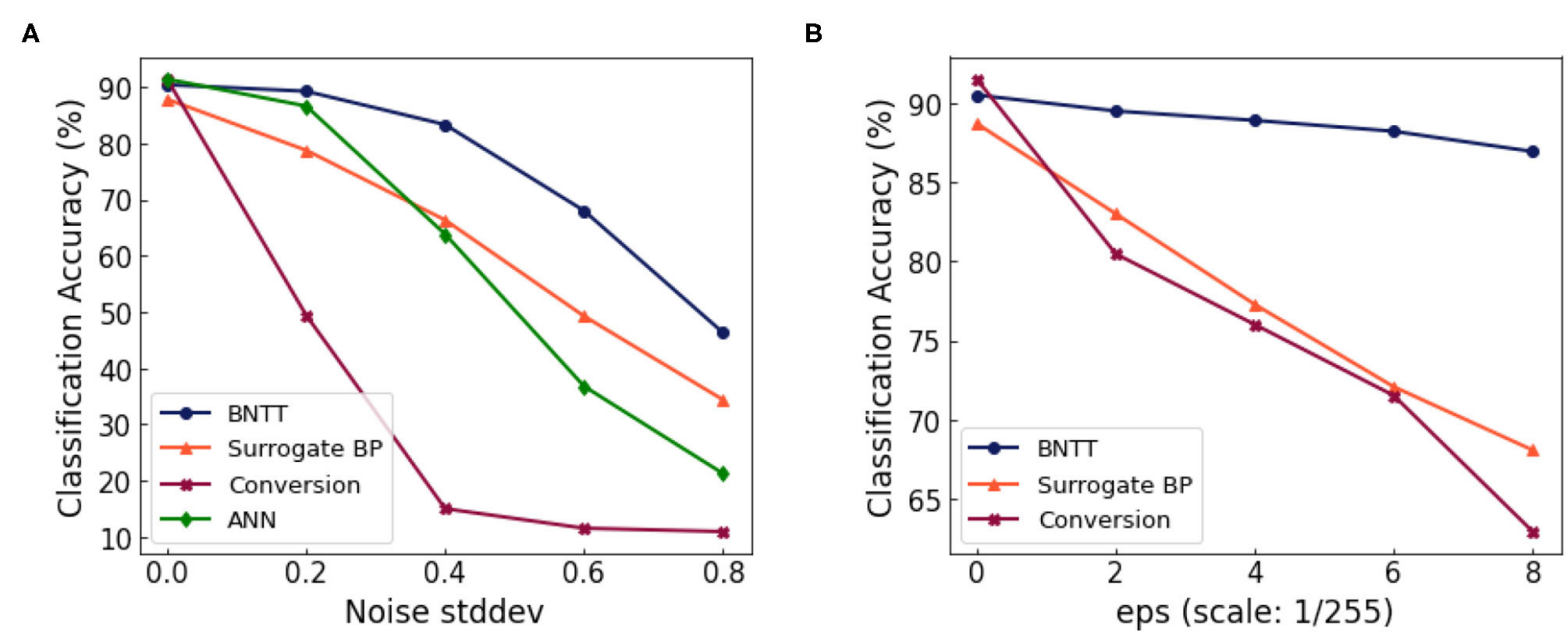
**(A)** Performance change with respect to the standard deviation of the Gaussian noise. **(B)** Performance change with respect to the attack intensity (ϵ, denoted in x-axis) of the FGSM attack.

Finally, we highlight the advantage of BNTT in terms of the robustness to noisy input. To investigate the effect of our BNTT for robustness, we evaluate the performance change in the SNNs as we feed in inputs with varying levels of noise. We generate the noisy input by adding Gaussian noise (0, σ^2^) to the clean input image. From Figure 8A, we observe the following: (i) The accuracy of conversion method degrades considerably for σ > 0.4. (ii) Compared to ANNs, SNNs trained with surrogate gradient back-propagation shows better performance at higher noise intensity. Still, they suffer from large accuracy drops in presence of noisy inputs. (iii) BNTT achieves significantly higher performance than the other methods across all noise intensities. This is because using BNTT decreases the overall number of time-steps which is a crucial contributing factor toward robustness (Sharmin et al., 2020). These results imply that, in addition to low-latency and energy-efficiency, our BNTT method also offers improved robustness for suitably implementing SNNs in a real-world scenario.

In order to further validate the robustness of BNTT, we conduct experiments on adversarial inputs. We use FGSM (Goodfellow et al., 2014) to generate adversarial samples for ANN. For a given image *x*, we compute the loss function L(x,y) with the ground truth label *y*. The objective of FGSM attack is to change the pixel intensity of the input image that maximizes the cost function:


(16) $$\begin{array}{l} x_{adv}=x+\epsilon \times sign\left(\nabla_{x}L\left(x,y\right)\right). \end{array}$$

We call *x*_*adv*_ as “adversarial sample.” Here, ϵ denotes the strength of the attack. To conduct the FGSM attack for SNN, we use the SNN-crafted FGSM method proposed in Sharmin et al. (2020). In Figure 8B, we show the classification performance for varying intensities of FGSM attack. The SNN approaches (e.g., BNTT and Surrogate BP) show more robustness than ANN due to the temporal dynamics and stochastic neuronal functionality. We highlight that our proposed BNTT shows much higher robustness compared to others. Thus, we assert that BNTT improves robustness of SNNs in addition to energy efficiency and latency.

### 4.8. Comparison With Layer Norm

Layer Normalization (LN) (Ba et al., 2016) is an optimization method for recurrent neural networks (RNNs). The authors asserted that directly applying BN layers is hardly applicable since RNNs vary with the length of the input sequence. To this end, an LN layer calculates the mean and the variance for every single layer. As SNNs also take time-sequence data as input, we compare our BNTT with Layer Normalization in Table 5. For all experiments, we use a VGG9 architecture. Also, we set a base learning rate to 0.3 and we use step-wise learning rate scheduling as described in section 4.1. The results show that BNTT is more suitable structure to capture the temporal dynamics of Poisson encoded spikes.

**Table 5:** Comparison with layer normalization on CIFAR-10 dataset.

**Method**	**Acc (%)**
Layer normalization (Ba et al., 2016)	75.4
BNTT	90.5

## 5. Conclusion

In this paper, we revisit the batch normalization technique and propose a novel mechanism for training low-latency, energy-efficient, robust, and accurate SNNs from scratch. Our key idea is to investigate the temporal characteristics of Batch Normalization (BN) with time-specific learnable parameters and statistics. Note, BN is known as an effective way of addressing vanishing/exploding gradients problem in ANNs. We discover that optimizing time-dependent learnable parameters γ captures the temporally varying input distribution so that it stabilizes the backward gradients during training and enables better learning of SNN representations. Our experiments reveal interesting benefits of BNTT for temporal early exit during inference as well as sturdy robustness against adversarial attacks. As previous SNN-based BN works (Fang et al., 2020; Ledinauskas et al., 2020; Zheng et al., 2020), this work showcases the importance of incorporating dynamic time-dependent parameters during surrogate gradient-based training to enable large-scale SNN implementations. By showing the importance of addressing the unstable gradient problem in SNN, we suggest future direction for better SNN training. Today, SNNs have few advanced optimization techniques (such as, weight initialization, skip connection that are common in ANN optimization suite) for addressing such issues. Our proposed BNTT can be considered to be one SNN-crafted optimization technique that can relieve the gradient problem, resulting in performance improvement. We hope this work fosters future work on advanced SNN optimization.

## Data Availability Statement

The original contributions presented in the study are included in the article/Supplementary Material, further inquiries can be directed to the corresponding author.

## Author Contributions

YK and PP conceived the work and contributed to the writing of the manuscript. YK carried out experiments. Both authors contributed to the article and approved the submitted version.

## Funding

This work was supported in part by the Center for Brain-inspired Computing (C-BRIC) which is a JUMP center sponsored by DARPA and SRC, the National Science Foundation (Grant#1947826), the Technology Innovation Institute, Abu Dhabi and the Amazon Research Award.

## Conflict of Interest

The authors declare that the research was conducted in the absence of any commercial or financial relationships that could be construed as a potential conflict of interest.

## Publisher's Note

All claims expressed in this article are solely those of the authors and do not necessarily represent those of their affiliated organizations, or those of the publisher, the editors and the reviewers. Any product that may be evaluated in this article, or claim that may be made by its manufacturer, is not guaranteed or endorsed by the publisher.
