# Supplementary material for: Revisiting Batch Normalization for Training Low-Latency Deep Spiking Neural Networks From Scratch
Source: Front Neurosci. 2021 Dec 9;15:773954. doi: 10.3389/fnins.2021.773954 (PMC8695433; doi:10.3389/fnins.2021.773954)
Supplement: Supplementary file 1 [file Data_Sheet_1.pdf]

Supplementary Material

A BACKWARD GRADIENT OF BNTT

Here, we calculate the backward gradient of a BNTT layer. Note that we omit the neuron index i for simplicity. For one sample x_b in a mini-batch, we compute the backward gradient of BNTT at time-step t:

$$\frac{\partial L}{\partial x_b^t} = \frac{\partial L}{\partial \hat{x}_b^t}\frac{\partial \hat{x}_b^t}{\partial x_b^t} + \frac{\partial L}{\partial \mu^t}\frac{\partial \mu^t}{\partial x_b^t} + \frac{\partial L}{\partial (\sigma^t)^2}\frac{\partial (\sigma^t)^2}{\partial x_b^t}. \tag{S1}$$

where,

$$\hat{x}_b^t = \frac{x_b^t - \mu^t}{\sqrt{(\sigma^t)^2 + \epsilon}}. \tag{S2}$$

It is worth mentioning that we accumulate input signals at the last layer in order to remove information loss. Then we convert the accumulated voltage into probabilities by using a softmax function. Therefore, we calculate backward gradient with respect to the loss L, following the previous work (Neftci et al., 2019). The first term of R.H.S in Eq. (S1) can be calculated as:

$$\frac{\partial L}{\partial \hat{x}_b^t}\frac{\partial \hat{x}_b^t}{\partial x_b^t} = \frac{1}{\sqrt{(\sigma^t)^2 + \epsilon}}\frac{\partial L}{\partial \hat{x}_b^t}. \tag{S3}$$

For the second term of R.H.S in Eq. (S1),

$$\begin{aligned}
\frac{\partial L}{\partial \mu^t}\frac{\partial \mu^t}{\partial x_b^t} &= \Big\{\frac{\partial L}{\partial \hat{x}^t}\frac{\partial \hat{x}^t}{\partial \mu^t} + \frac{\partial L}{\partial (\sigma^t)^2}\frac{\partial (\sigma^t)^2}{\partial \mu^t}\Big\}\frac{\partial \mu^t}{\partial x_b^t} \\
&= \Big\{\sum_{j=1}^{m}\frac{\partial L}{\partial \hat{x}_j^t}\frac{-1}{\sqrt{(\sigma^t)^2 + \epsilon}} + \frac{\partial L}{\partial (\sigma^t)^2}\frac{1}{m}\sum_{j=1}^{m} -2(x_j^t - \mu^t)\Big\}\frac{\partial \mu^t}{\partial x_b^t} \\
&= \Big\{\sum_{j=1}^{m}\frac{\partial L}{\partial \hat{x}_j^t}\frac{-1}{\sqrt{(\sigma^t)^2 + \epsilon}} - 2\frac{\partial L}{\partial (\sigma^t)^2}(\mu^t - \frac{\mu^t m}{m})\Big\}\frac{\partial \mu^t}{\partial x_b^t} \\
&= \Big\{\sum_{j=1}^{m}\frac{\partial L}{\partial \hat{x}_j^t}\frac{-1}{\sqrt{(\sigma^t)^2 + \epsilon}}\Big\}\frac{\partial \mu^t}{\partial x_b^t} \\
&= \frac{1}{m\sqrt{(\sigma^t)^2 + \epsilon}}\Big\{-\sum_{j=1}^{m}\frac{\partial L}{\partial \hat{x}_j^t}\Big\}.
\end{aligned} \tag{S4}$$

For the third term of R.H.S in Eq. (S1),

$$\begin{aligned}
\frac{\partial L}{\partial(\sigma^t)^2}\frac{\partial(\sigma^t)^2}{\partial x_b^t} &= \Big\{\frac{\partial L}{\partial \hat{x}^t}\frac{\partial \hat{x}^t}{\partial(\sigma^t)^2}\Big\}\frac{\partial(\sigma^t)^2}{\partial x_b^t} \\
&= \Big\{-\frac{1}{2}\sum_{j=1}^{m}\frac{\partial L}{\partial \hat{x}_j^t}(x_j^t-\mu^t)((\sigma^t)^2+\epsilon)^{-1.5}\Big\}\frac{\partial(\sigma^t)^2}{\partial x_b^t} \\
&= \Big\{-\frac{1}{2}\sum_{j=1}^{m}\frac{\partial L}{\partial \hat{x}_j^t}(x_j^t-\mu^t)((\sigma^t)^2+\epsilon)^{-1.5}\Big\}\frac{2(x_b-\mu^t)}{m} \\
&= \Big\{-\sum_{j=1}^{m}\frac{\partial L}{\partial \hat{x}_j^t}\frac{(x_j^t-\mu^t)}{\sqrt{(\sigma^t)^2+\epsilon}}((\sigma^t)^2+\epsilon)^{-0.5}\Big\}\frac{(x_b-\mu^t)}{m\sqrt{(\sigma^t)^2+\epsilon}} \\
&= \Big\{-\sum_{j=1}^{m}\frac{\partial L}{\partial \hat{x}_j^t}\hat{x}_j^t((\sigma^t)^2+\epsilon)^{-0.5}\Big\}\frac{\hat{x}_b}{m} \\
&= \frac{\hat{x}_b}{m\sqrt{(\sigma^t)^2+\epsilon}}\Big\{-\sum_{j=1}^{m}\frac{\partial L}{\partial \hat{x}_j^t}\hat{x}_j^t\Big\}.
\end{aligned} \tag{S5}$$

Based on Eq (S3), Eq (S4), and Eq (S5), we can reformulate Eq. (S1) as:

$$\begin{aligned}
\frac{\partial L}{\partial x_b^t} &= \frac{\partial L}{\partial \hat{x}_b^t}\frac{\partial \hat{x}_b^t}{\partial x_b^t} + \frac{\partial L}{\partial \mu^t}\frac{\partial \mu^t}{\partial x_b^t} + \frac{\partial L}{\partial(\sigma^t)^2}\frac{\partial(\sigma^t)^2}{\partial x_b^t} \\
&= \frac{1}{\sqrt{(\sigma^t)^2+\epsilon}}\frac{\partial L}{\partial \hat{x}_b^t} + \frac{1}{m\sqrt{(\sigma^t)^2+\epsilon}}\Big\{-\sum_{j=1}^{m}\frac{\partial L}{\partial \hat{x}_j^t}\Big\} \\
&\quad + \frac{\hat{x}_b}{m\sqrt{(\sigma^t)^2+\epsilon}}\Big\{-\sum_{j=1}^{m}\frac{\partial L}{\partial \hat{x}_j^t}\hat{x}_j^t\Big\} \\
&= \frac{1}{m\sqrt{(\sigma^t)^2+\epsilon}}\Big\{m\frac{\partial L}{\partial \hat{x}_b^t} - \sum_{j=1}^{m}\frac{\partial L}{\partial \hat{x}_j^t} - \hat{x}_b\sum_{j=1}^{m}\frac{\partial L}{\partial \hat{x}_j^t}\hat{x}_j^t\Big\}.
\end{aligned} \tag{S6}$$

To summarize, for every time-step t, gradients are calculated based on the time-specific statistics of input signals. This allows the networks to take into account temporal dynamics for training weight connections.

B RATE CODING

Spiking neural networks process multiple binary spikes. Therefore, for training and inference, a static image needs to be converted. There are various spike coding schemes such as rate, temporal, and phase (Mostafa, 2017; Kim et al., 2018). Among them, we use rate coding due to its reliable performance across various tasks. Rate coding provides spikes proportional to the pixel intensity of the given image. In order to implement this, following previous work (Roy et al., 2019), we compare each pixel value with a random number ranging between $[I_{min}, I_{max}]$ at every time-step. Here, I_{min}, I_{max} correspond to the minimum and maximum possible pixel intensity. If the random number is greater than the pixel intensity, the Poisson

spike generator outputs a spike with amplitude 1. Otherwise, the Poisson spike generator does not yield any spikes.

C DVS-CIFAR10 DATASET

On DVS-CIFAR10, following (Wu et al., 2019), we downsample the size of the 128 ×128 images to 42×42. Also, we divide the total number of time-steps available from the original time-frame data into 20 intervals and accumulate the spikes within each interval. We use a similar architecture as previous work (Wu et al., 2019), which consists of a 5-layerered feature extractor and a classifier. The detailed architecture is shown in Fig. S1 in this appendix.

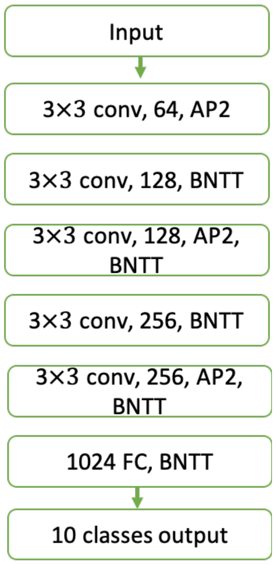

Figure S1: Illustration of network structures for DVS dataset. Here, AP denotes average pooling, FC denotes fully connected configuration.

D SPIKE RATE CALCULATION

In this appendix section, we provide the details of energy calculation discussed in Section 4.3 in the main paper. The total computational cost is proportional to the total number of floating point operations (FLOPS). This is approximately the same as the number of Matrix-Vector Multiplication (MVM) operations. For layer l in ANNs, we can calculate FLOPS as:

$$FLOPS_{ANN}(l) = \begin{cases} k^2 \times O^2 \times C_{in} \times C_{out}, & \text{if } l\text{: Conv,} \\ C_{in} \times C_{out}, & \text{if } l\text{: Linear.} \end{cases} \tag{S7}$$

Here, k is kernel size. O is output feature map size. C_{in} and C_{out} are input and output channels, respectively. For SNNs, we first define spiking rate $R_s(l)$ at layer l which is the average firing rate per neuron.

$$R_s(l) = \frac{\#\text{spikes of layer } l \text{ over all timesteps}}{\#\text{neurons of layer } l}. \tag{S8}$$

Table S1. Normalized energy comparison on neuromorphic architecture: TrueNorth(Akopyan et al., 2015). We set conversion as a reference for normalized energy comparison. We conduct experiments on CIFAR-10 with a VGG9 architecture.

Method	Time-steps	#Spikes (10^4)	Energy (Akopyan et al., 2015)
Conversion	1000	419.30	1
Surrogate	100	141.96	0.3384
BNTT	25	13.106	0.0312

E ENERGY COMPARISON IN NEUROMORPHIC ARCHITECTURE

We further show the energy-efficiency of BNTT in a neuromorphic architecture, TrueNorth (Akopyan et al., 2015). Following the previous work (Park et al., 2020; Moradi and Manohar, 2018), we compute the normalized energy, which can be classified into dynamic energy (E_{dyn}) and static energy (E_{sta}). The E_{dyn} value corresponds to the computing cores and routers, and E_{sta} is for maintaining the state of the CMOS circuit. The total energy consumption can be calculated as #Spikes $\times$ E_{dyn} + #Time-step $\times$ E_{sta}, where (E_{dyn}, E_{sta}) are (0.4, 0.6). In Table S1, we show that our BNTT has a huge advantage in terms of energy efficiency in neuromorphic hardware.

REFERENCES

Akopyan, F., Sawada, J., Cassidy, A., Alvarez-Icaza, R., Arthur, J., Merolla, P., et al. (2015). Truenorth: Design and tool flow of a 65 mw 1 million neuron programmable neurosynaptic chip. *IEEE transactions on computer-aided design of integrated circuits and systems* 34, 1537–1557

Kim, J., Kim, H., Huh, S., Lee, J., and Choi, K. (2018). Deep neural networks with weighted spikes. *Neurocomputing* 311, 373–386

Moradi, S. and Manohar, R. (2018). The impact of on-chip communication on memory technologies for neuromorphic systems. *Journal of Physics D: Applied Physics* 52, 014003

Mostafa, H. (2017). Supervised learning based on temporal coding in spiking neural networks. *IEEE transactions on neural networks and learning systems* 29, 3227–3235

Neftci, E. O., Mostafa, H., and Zenke, F. (2019). Surrogate gradient learning in spiking neural networks. *IEEE Signal Processing Magazine* 36, 61–63

Park, S., Kim, S., Na, B., and Yoon, S. (2020). T2fsnn: Deep spiking neural networks with time-to-first-spike coding. *arXiv preprint arXiv:2003.11741*

Roy, K., Jaiswal, A., and Panda, P. (2019). Towards spike-based machine intelligence with neuromorphic computing. *Nature* 575, 607–617

Wu, Y., Deng, L., Li, G., Zhu, J., Xie, Y., and Shi, L. (2019). Direct training for spiking neural networks: Faster, larger, better. In *Proceedings of the AAAI Conference on Artificial Intelligence*. vol. 33, 1311–1318
